# Back to bedside: Renal point-of-care ultrasonography (POCUS) by internal medicine resident physicians for identification of hydronephrosis in patients with acute kidney injury

**DOI:** 10.5339/qmj.2025.36

**Published:** 2025-06-11

**Authors:** Fateen Ata, Rohit Sharma, Jaweria Akram, Bashar Tanous, Abdulla Arshad, Mohammed Alamin, Abdulrahman Al-Mashdali, Zohaib Yousaf, Muhammad Zahid

**Affiliations:** 1 Department of Endocrinology and Metabolism, Hamad Medical Corporation, Doha, Qatar; 2 Department of Internal Medicine, Mass General Brigham, Boston, MA, USA; 3 Department of Critical Care Medicine, Hamad Medical Corporation, Doha, Qatar; 4 Department of Internal Medicine, University of California San Francisco, Fresno, CA, USA; 5 Department of Pulmonary Medicine, Hamad Medical Corporation, Doha, Qatar; 6 Department of Internal Medicine, Hamad Medical Corporation, Doha, Qatar; 7 Department of Oncology, Hamad Medical Corporation, Doha, Qatar; 8 Department of Internal Medicine, Reading Hospital, Tower Health, PA, USA *Email: rsharma25@mgb.org

**Keywords:** Point-of-care ultrasound, POCUS, acute kidney injury, hydronephrosis, internal medicine resident

## Abstract

**Background:**

Point-of-care ultrasonography (POCUS) has rapidly emerged as a valuable diagnostic tool in various medical conditions, including acute kidney injury (AKI) in the Western healthcare system. Its utility in the Middle East and Asian healthcare setups remains under-explored. This study aimed to assess the effectiveness of POCUS, performed by internal medicine residents (IMRs), in diagnosing hydronephrosis in AKI patients at a tertiary care training hospital in Qatar.

**Methods:**

We conducted a pilot prospective cross-sectional study from June 2021 to September 2021, enrolling adult patients admitted with AKI in the acute medical assessment unit (AMAU) via convenience sampling. IMRs received mandatory POCUS training (including a 30-minute didactic teaching session and supervised performance of renal POCUS scans). The primary outcome was the detection of hydronephrosis, with findings compared to departmental renal ultrasound scans performed by the radiologists.

**Results:**

Fifty patients were included, with POCUS identifying hydronephrosis in five out of nine patients with confirmed hydronephrosis via official departmental renal ultrasound, demonstrating a sensitivity of 83.3% and specificity of 93% for POCUS performed by IMRs. Hydronephrosis via bedside POCUS scans had clinically reliable positive and negative predictive values (55.6% and 98%, respectively). Cohen’s kappa was 0.7 (0.45–0.94), indicating substantial agreement. One patient whose renal POCUS was reported as normal by IMR was identified to have hydronephrosis on an official departmental renal ultrasound.

**Conclusion:**

This study demonstrated the effectiveness of adequate training in improving the diagnostic skills of residents using POCUS for bedside detection of hydronephrosis in patients with AKI in a residency program from the Middle East. Residency programs that include POCUS training have the potential to significantly increase bedside diagnostic capabilities with improved quality of training and patient care.

## Background

Point-of-care ultrasonography (POCUS), initially introduced in 1998 as a military tool and considered a potential threat to traditional radiology practices because of concerns related to quality, training, regulatory standards, and economic impact, has emerged as an extremely valuable adjunct to bedside clinical examinations.^1,2^ It is rapidly establishing as a standard-of-care tool in diagnosing and managing various medical conditions.^3^ Around one-third of US medical schools have incorporated POCUS teaching in their curricula, and the trend is bound to increase in the coming years.^4,5^ However, the utility of POCUS by medical students and trainees is yet to globalize.

One area where the utility of POCUS by medical trainees is of particular interest is the bedside assessment of kidneys.^6^ Postrenal acute kidney injury (AKI), which occurs due to the obstruction of urinary flow, accounts for 5%–10% of all AKI cases.^7^ Given its potential for reversibility, prompt diagnosis is critical. Physical examination alone cannot diagnose postrenal AKI; therefore, imaging is necessary for confirmation. Ultrasonography is the preferred modality to detect hydronephrosis, a key indicator of postrenal AKI.^7,8^

In AKI, bedside POCUS may facilitate early identification of hydronephrosis indicative of obstructive nephropathy as the underlying etiology, thereby enabling timely diagnosis, expediting therapeutic management, and decreasing fragmentation of care.^9^ Incorporating renal POCUS training in the internal medicine residency program allows residents to develop new skills for day-to-day clinical practice and enhance bedside assessment of patients with AKI. Additionally, data has shown a multitude of healthcare-related benefits with the accurate use of POCUS in the assessment of AKI, including a reduced hospital length of stay, decreased healthcare-related cost burden, better clinical care, and less patient discomfort.^10^ Multiple studies have shown variable sensitivity (72%–96%) and specificity (59%–96%) in detecting hydronephrosis by POCUS by trainees of various specialties.^10^ These studies come mainly from Western countries, and limited data is available with some barriers to the utility of POCUS in detecting hydronephrosis by trainees in other parts of the world.^11–13^ We aimed to assess the effectiveness of POCUS in detecting hydronephrosis from a tertiary care training hospital in Qatar by trainee medical residents.

## Methods

We conducted a pilot prospective cross-sectional study at Hamad General Hospital, the largest public tertiary care hospital in Doha, Qatar. Adult patients admitted to the acute medical assessment unit (AMAU) and medical wards for evaluation of AKI between June 2021 and September 2021 were selected via convenience sampling. For this study, hydronephrosis was defined as the dilation or swelling of one or both kidneys due to a build-up of urine.^14^ For patient enrollment, the AKI definition by the Kidney Disease: Improving Global Outcomes (KDIGO) was used: “a rise in creatinine of at least 26.5 μmol/L within 48 hours or by 50% from baseline within last 7 days, or a urine volume of <0.5 mL/kg/hours for at least 6 hours”.^15^ Patients with known chronic kidney disease or patients on dialysis were excluded from the study. This study was classified as a service improvement project by the Institutional Review Board (IRB) of Hamad Medical Corporation. Therefore, it did not require a formal IRB review or approval.

A total of five IMRs participated in this study. Each resident was tasked with performing renal POCUS on 10 patients, resulting in a total of 50 patients being included in the study. Each patient was screened by a single IMR to ensure consistency and minimize variability in the ultrasound findings. IMRs received mandatory training via a 30-minute didactic teaching session by a certified attending level physician and performance of at least ten renal POCUS scans under direct one-to-one supervision to ensure efficient technique and evaluation of appropriate ultrasound findings. The residents were taught basic principles of ultrasound physics (sound wave generation, propagation, reflection, and attenuation), detailed renal anatomy, how to pick up dilation of the renal pelvis, detect the presence of fluid in the kidneys, calyceal dilation, and thinning of the renal parenchyma. The same POCUS machine was used for teaching and study to avoid machine-to-machine variability and bias. The IMRs included in the study did not have prior formal training in POCUS use. A baseline assessment of each IMR’s POCUS skills was conducted prior to training to measure improvement. Posttraining evaluations were done to ensure all IMRs achieved the required level of proficiency before participating in the study. Patients admitted to the AMAU who fulfilled the inclusion criteria were informed that a POCUS would be conducted as part of their bedside assessment to aid in the early identification of hydronephrosis. Patients were informed that the POCUS would be performed by IMRs who had undergone a structured training protocol, including a 30-minute didactic session encompassing ultrasound physics, renal anatomy, and hydronephrosis detection. Verbal consent was obtained from all patients, and it was explained that the POCUS findings would be confirmed by a formal departmental renal ultrasound, ensuring the standard-of-care provision.

IMRs performed bedside POCUS to assess hydronephrosis as the primary outcome, and findings were compared with departmental renal ultrasound scans performed prospectively by the radiology team. The ultrasound technician conducting the departmental scans and the radiologists reporting the scans were blinded to the bedside POCUS findings to eliminate reporting bias. The POCUS findings by the residents were submitted to the data collector before the departmental ultrasound and were not subject to any amendment thereafter.

## Results

A total of 50 patients were included in the study, all of whom had provisional diagnoses of AKI based on clinical presentation and deranged renal parameters, pending official departmental renal ultrasound scans. Hydronephrosis was identified in nine patients on bedside POCUS, of which one patient had bilateral hydronephrosis, while eight had unilateral findings of hydronephrosis. Out of these nine patients, hydronephrosis was confirmed in five patients on official departmental renal ultrasound scans. On the other hand, one patient whose renal POCUS was reported normal was identified to have hydronephrosis on an official departmental renal ultrasound (Table 1). The single false negative case was mild hydronephrosis. The false positive cases were 4, detected as mild hydronephrosis by the residents; later, the official departmental ultrasound showed no hydronephrosis. Cohen’s kappa statistics were applied to measure the level of agreement between POCUS done by IMRs and departmental renal ultrasound. Unweighted kappa was found to be κ = 0.7 (0.45–0.94). This indicates substantial agreement between the two techniques (bedside POCUS by residents and departmental ultrasound).

## Discussion

This study showed that the IMRs can accurately identify hydronephrosis in patients with AKI with appropriate training. POCUS performed at the time of admission showed good sensitivity (83.3%) and specificity (93%) with clinically reliable positive and negative predictive values (55.6% and 98%, respectively). Cohen’s kappa statistics revealed a substantial agreement with the POCUS done by IMRs [κ = 0.7 (0.45–0.94)].

AKI is a common and serious clinical event associated with increased short- and long-term mortality, as well as the development of chronic kidney disease. At a given time, approximately 15% of hospitalized patients and 50% of those admitted to intensive care units (ICUs) have AKI.^16,17^ The diagnosis of prerenal and postrenal injuries is vital as timely intervention prevents intrinsic renal injury. The National Institute of Clinical Excellence (NICE) recommends an ultrasound (USG) of the urinary tract for patients with AKI with no apparent cause identified or at risk of urinary obstruction within 24 hours.^18^

Bedside POCUS examination is rapidly gaining evidence to support its use to aid traditional examination techniques in diagnosing and managing acutely unwell adult patients.^19^ The residents serve as patients’ primary point of contact, offering immediate care regardless of the time. POCUS would be a valuable tool for the internal medicine registrar to use to deliver efficient, effective, and timely care. Training programs like intensive care and emergency medicine have already incorporated POCUS in their training curriculum. Society for Acute Medicine, UK, published a focused acute medicine ultrasound (FAMUS) curriculum, and the Canadian Internal Medicine Ultrasound group has designed the POCUS internal medicine curriculum covering essential areas in managing acutely unwell patients.^20–22^ Although POCUS improves diagnostic accuracy and reduces the time to diagnosis, whether it makes a difference in patient care remains to be determined. Evidence suggests a better doctor-patient relationship and patient satisfaction with care from POCUS use.^23–25^

The concordance between POCUS findings and official ultrasounds in various medical conditions highlights POCUS’s potential and universal acceptability as a fairly reliable, rapid, first-line diagnostic tool, offering a swift, accurate glimpse into patient health issues.^26^ While our study did not prespecify measuring the time taken to diagnose hydronephrosis on POCUS compared to the official departmental ultrasound, reports from POCUS were always significantly ahead in time compared to official ultrasounds. This highlights that POCUS, when performed by properly trained residents, may serve as an important and time-efficient alternative to traditional departmental ultrasound for diagnosing conditions like hydronephrosis immediately at the first patient encounter.^27^

Many studies are emerging advocating the utility of POCUS in AKI. Parulekar et al. looked into the performance of POCUS in patients with AKI in the ICU and demonstrated accuracy with bedside imaging, with the single patient who had hydronephrosis correctly picked up by POCUS.^28^ Sibley et al. conducted a larger study, including 413 patients with renal colic, showing a specificity of 71.8% and sensitivity of 77.1% for hydronephrosis detection via POCUS [29]. In our study, hydronephrosis was present in 9 out of 50 patients (18%), with POCUS correctly identifying 5 out of the 9 departmentally confirmed cases, resulting in 4 false positives and 1 false negative. In contrast, the study by Sibley et al. reported hydronephrosis in 51% of patients on departmental imaging (211 out of 413) and in 53% of patients on POCUS (219 out of 413), having 48 false negatives and 57 false positives. The differences in detection rates can be attributed to different sample sizes, patient populations (patients with AKI of any cause in our study vs. an obstructive cause in the latter study), clinical settings, and operator training levels.^29^

Another quality improvement study by Nepal et al. provides compelling evidence for the utility of POCUS in the acute medical setting, demonstrating a sensitivity of 90% and a specificity of 100% for diagnosing obstructive uropathy in patients with AKI.^30^ In 54 patients, performers could identify hydronephrosis in 9 kidneys (8.6% of total scans), matched by departmental scan findings. The authors also reported that the median time of POCUS examination was 6 minutes, with a median reporting time of 10 minutes, highlighting its time effectiveness in clinical settings.^30^ Javudin et al. published a study in 2017 in which emergency medicine physicians with no prior POCUS skills were provided with brief training (a 16-hour training program over 2 days). This translated into an impressive 100% sensitivity and a specificity of 71% for the diagnosis of hydronephrosis.^31^ Hence, more and more evidence is now accumulating that even limited training with POCUS can produce appreciable results in point-of-care diagnostic capabilities for common medical conditions, saving critical time and healthcare costs and simultaneously improving the quality of life for the patients.^32^ Findings from our study align with those from Caronia et al., who reported substantial agreement with departmental sonograms and, likewise, high sensitivity and specificity.^33^ Training in the latter study was relatively shorter (5-hour module). Yet, the results were impressive and promising in favor of routine use of POCUS by medicine residents for disease diagnosis and prognostication.

Although most studies advocating the use of POCUS in detecting hydronephrosis showed positive results, variability in results was noted. These differences can be due to variable etiologies underlying the AKI, different patient populations, machine-to-machine variability, different inpatient settings (medical versus critical care), and differences in the POCUS teaching methods. One of the key points is the standardization of POCUS training in internal medicine in the shape of structured education within the residency programs.^34^ The variability in hydronephrosis detection rates between our study and others highlights operator proficiency’s important role in utilizing POCUS effectively.^34^

Our study had various limitations which might be imperative to exploratory quality improvement study designs. We utilized convenience sampling to select patients, minimizing the generalizability of the findings of our study. The study being conducted in a single tertiary care center with a unified academic curriculum may limit the applicability of the results to other healthcare settings or regions with varying levels of resources and training in POCUS. Despite adequate training by experts, operator-dependency of POCUS coupled with variability in the skill level among residents could potentially influence uniformity in diagnostic accuracy. Our sample size included only 50 patients, which might not be large enough to establish POCUS’s efficacy and reliability in detecting hydronephrosis across a broader patient population. Another limitation of our study is the relatively small number of IMRs (five) performing the scans, limiting the generalizability of our findings. Additionally, while perceived levels of confidence and competence in performing POCUS scans were assessed through pretraining and posttraining POCUS scans, this data was not formally analyzed, limiting the quantitative evaluation of the training program’s effectiveness in improving residents’ confidence in the use of POCUS. Lastly, reliance on departmental ultrasounds as the gold standard for diagnostic comparison could introduce bias as the scans were not performed consistently by the same technician and reported by the same radiologist. Despite the acknowledged limitations, our study has many strengths. One strength of this study is that we provided our residents with supervised and well-structural training, with the end-of-training certification by a certified POCUS specialist. This 30-minute didactic session and supervised hands-on practice was more extensive than many sessions reported in previous similar studies. The main barriers to the widespread use of POCUS are the availability of ultrasound machines, well-designed training curricula, and well-trained educators. Additionally, there is an ad hoc approach at the undergraduate and postgraduate level regarding POCUS teaching and training, which relies primarily on the goodwill of trainers to deliver training to a select few. There is ample opportunity to develop a POCUS curriculum and structured training program. This study is one of the pioneer studies in the region and will promote further studies from the Middle Eastern and South Asian regions where POCUS utilization is still limited.

## Conclusion

Structured POCUS training enhances residents’ skills in diagnosing hydronephrosis in patients with AKI, showing high diagnostic accuracy and the potential to significantly enhance the quality of patient care. The high sensitivity (83.3%) and specificity (93%) achieved in the detection of hydronephrosis via POCUS indicate that adequately trained IMRs can effectively use POCUS as a reliable diagnostic tool, leading to timely and accurate patient management. Our pilot study lays the groundwork for future, more elaborate research on POCUS in AKI and other medical conditions from the Middle East, advocating for the inclusion of POCUS training in medical education. The authors fully support incorporating POCUS training in the internal medicine training curriculum.

## Conflicts of interest

None.

## Figures and Tables

**Table 1.** Diagnostic accuracy of bedside POCUS for diagnosis of hydronephrosis in patients with AKI by internal medicine residents (IMR).

**Official departmental renal ultrasound**			
**IMR bedside renal POCUS**	**Hydronephrosis present**	**Hydronephrosis absent**	
Hydronephrosis present	5	4	PPV: 55.6% (5/9)
Hydronephrosis absent	1	54	NPV: 98% (54/55)
	Sensitivity: 83.3% (5/6)	Specificity: 93% (54/58)	

POCUS: point-of-care ultrasound, IMR: internal medicine resident, PPV: positive predictive value, NPV: negative predictive value, AKI: acute kidney injury.
